# Minoxidil induced central serous Chorioretinopathy treated with oral Eplerenone – a case report

**DOI:** 10.1186/s12886-020-01499-6

**Published:** 2020-06-05

**Authors:** Ramesh Venkatesh, Arpitha Pereira, Kushagra Jain, Naresh Kumar Yadav

**Affiliations:** Department of Retina & Vitreous, Narayana Nethralaya, 121/C, Chord Road, 1st ‘R’ Block, Rajaji Nagar, Bengaluru, 560010 India

**Keywords:** Central serous chorioretinopathy, Eplerenone, Minoxidil, Case report

## Abstract

**Background:**

Minoxidil solution has routinely been used for decades for the treatment of androgenic alopecia. Central serous chorioretinopathy (CSCR) is a rare side-effect noted following prolonged topical minoxidil therapy for androgenic alopecia. In this report, we describe a case of a 41-year-old young man who developed CSCR following prolonged therapy with topical Minoxidil solution and was treated with oral eplerenone.

**Case presentation:**

A 41-year-old male presented to the retina clinic with complaints of seeing a black spot, blurred vision and metamorphopsia involving the right eye for the past 4 months. He was on treatment for androgenic alopecia with topical 5% Minoxidil application on scalp two times a day. He noticed the symptoms 8 months after starting the treatment and had stopped the medication since the past 2 months. On examination, best-corrected visual acuity was 20/20 in both eyes. Fundoscopic examination of the right eye with +78D lens on slit lamp revealed the presence of subretinal fluid and few focal spots of retinal pigment epithelial alterations. Optical coherence tomography scan evaluation showed the presence of subretinal fluid (SRF) and pachychoroid supporting the diagnosis of CSCR. Indocyanine green angiography revealed dilated hyperpermeable choroidal vasculature on the nasal side of the fovea in the early and later phases of the angiogram. The patient was diagnosed with CSCR as a possible consequence of the topical minoxidil solution. Patient was asked to avoid future use of Minoxidil and was started on oral eplerenone therapy 50 mg/day for 4 consecutive weeks. One month later, there was complete resolution of his symptoms and SRF. At the final follow-up visit, 2 months after starting the therapy, there was no recurrence of SRF.

**Conclusion:**

CSCR is a rare side-effect noted following prolonged topical minoxidil therapy for androgenic alopecia. While we found oral eplerenone to be safe and effective, further studies would be required before it can be routinely used in the population.

## Background

Since the last three decades, Minoxidil has been used as an effective treatment option in androgenic alopecia. However, its mechanism of action is not well understood. Studies in animals have shown that topical minoxidil affects the hair growth by shortening the telogen phase and causing premature entry of resting hair follicles into anagen phase [1, 2] Minoxidil probably has a similar action in humans as well. Minoxidil via its activated sulphated metabolite, Minoxidil sulphate causes K-ATP channels opening and vascular smooth muscle relaxation. In addition, minoxidil produces growth factors like vascular endothelial growth factor (VEGF) which promote hair growth [3–6] The common adverse reactions of topical minoxidil formulation are limited to irritant and allergic contact dermatitis on the scalp [7] The local side effects following minoxidil therapy are dependent on the contact time of applied dose, concentration and percutaneous absorption of the drug [8] Ocular side-effects following topical minoxidil usage have rarely been reported [9, 10] In this report, we describe a rare ocular side effect of central serous chorioretinopathy (CSCR) developing after prolonged usage of 5% topical minoxidil solution which was treated with oral eplerenone.

## Case presentation

**Fig. 1:**
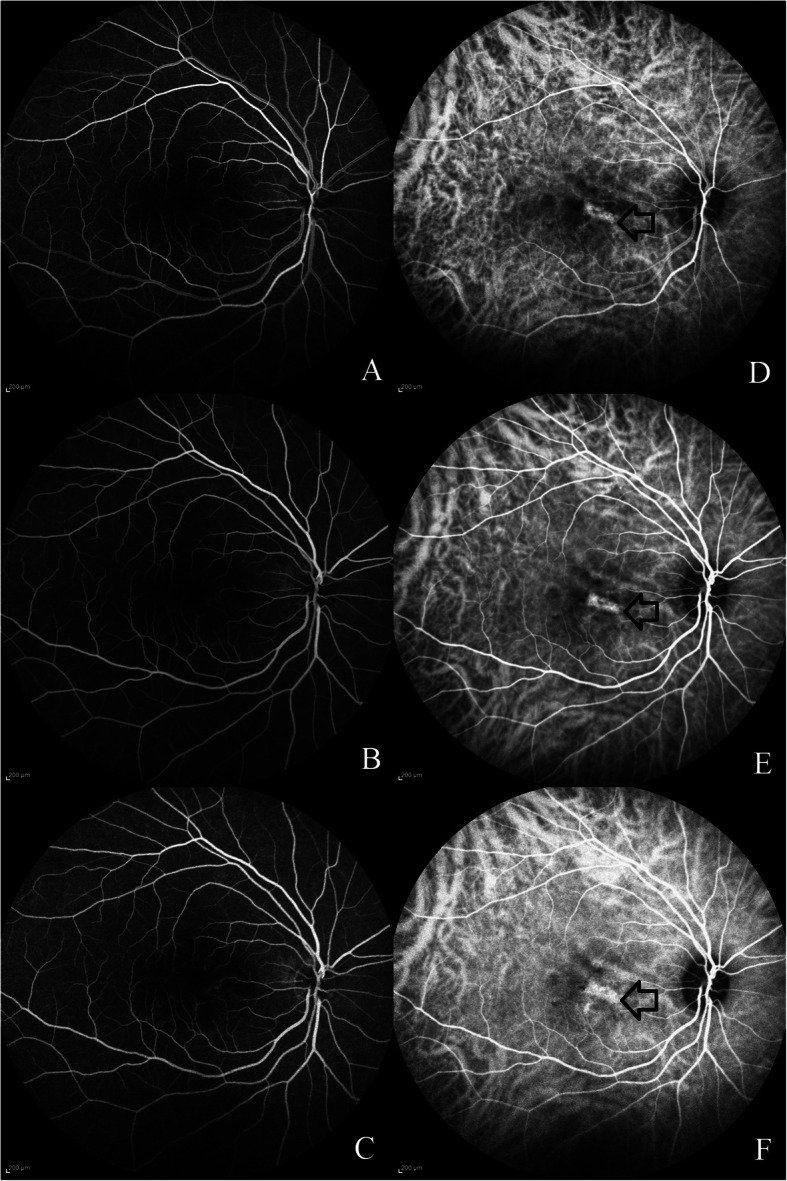
Fluorescein (FA) and indocyanine green (ICGA) angiography imaging features: Fig. 1**a-c**: Progressive phases of the fluorescein angiography did not show the classic smoke stack or ink-blot pattern of leakage. Figure 1**d-f**: Progressive phases of indocyanine green angiography shows the dilated hyperpermeable choroidal vessel nasal to the fovea (black arrow)

**Fig. 2:**
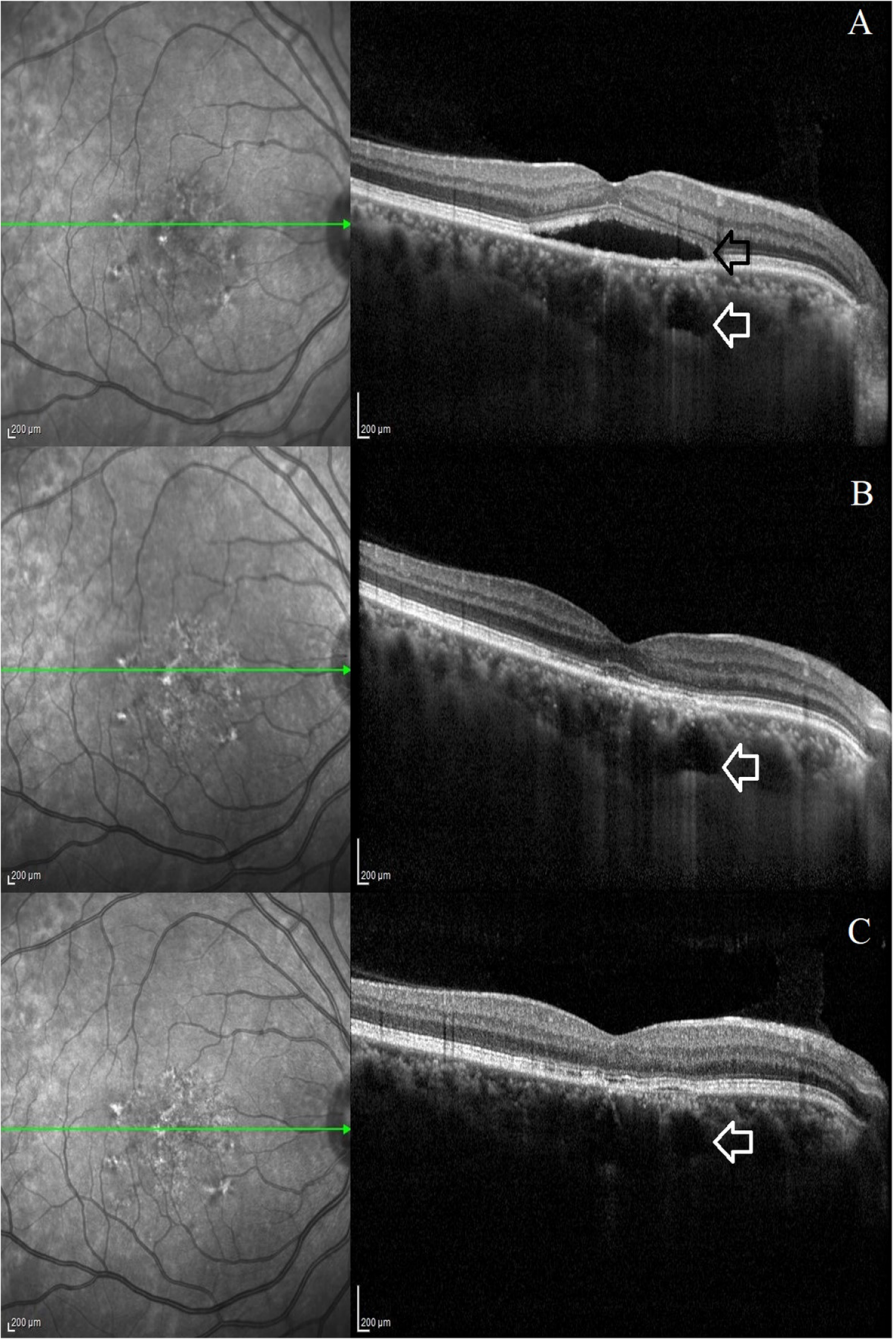
Pre- and post-treatment optical coherence tomography (OCT) imaging findings following therapy with oral eplerenone. Figure 2**a**: This picture shows the subretinal fluid at the macula with irregular retinal pigment epithelial changes noted nasal to fovea (black arrow). There is increased choroidal thickness with presence of dilated pachy choroidal vessel nasal to fovea (white arrow). Figure 2**b**: OCT scan at 1-month shows complete subretinal fluid resolution following therapy with oral eplerenone. Figure 2**c**: OCT scan at 2-month shows no subretinal fluid after stopping oral eplerenone

A 41-year-old young healthy male entered the retina clinic with complaints of seeing a black spot, blurred vision and metamorphopsia involving the right eye for the past 4 months. He was on treatment for androgenic alopecia with topical 5% Minoxidil application on scalp two times a day for a total time period of 10 months. He noticed the symptoms 8 months after starting the treatment. He was not seen by any other ophthalmologist previously and had stopped the medication on his own since the past 2 months. The patient denied using other medications or a history of previous treatment with corticosteroids. On examination, best-corrected visual acuity was 20/20 in both eyes. Intraocular pressure was 15 mmHg in both eyes. Anterior segment was unremarkable. Fundoscopic examination of the right eye with +78D lens on slit lamp revealed central swelling located over the macula with presence of subretinal fluid (SRF) and few focal spots of retinal pigment epithelial alterations. Left eye fundus was normal. Optical coherence tomography (OCT) scan evaluation showed the presence of SRF with an irregular retinal pigment epithelium. On enhanced depth imaging OCT, dilated pachy choroidal vessels compressing the overlying Sattler’s and choriocapillaris layer was noted nasal to the fovea. Subfoveal choroidal thickness measured was 425 μm. Fluorescein angiography did not show any classic smoke stack or ink-blot pattern of leaks. Indocyanine green angiography (ICGA) revealed dilated hyperpermeable choroidal vasculature on the nasal side of the fovea in the early and later phases of the angiogram corresponding to the pachy choroidal vessels seen on enhanced depth imaging OCT (Fig. 1). The patient was diagnosed with CSCR as a possible consequence of his topical minoxidil solution. Patient was asked to avoid future use of Minoxidil and was started on oral eplerenone therapy 50 mg/day for 4 consecutive weeks. Serum electrolytes and renal profile were done prior to starting the therapy. Blood investigations were normal. One month later, he was found to have complete resolution of his symptoms. OCT examination revealed complete resolution of SRF and reduction in the subfoveal choroidal thickness (391 μm). Oral eplerenone was discontinued and patient was asked to follow up the next month. No side-effects were reported following the intake of oral eplerenone. At the final follow-up visit, 2 months after starting the therapy, there was no recurrence of SRF and the subfoveal choroidal thickness further decreased to 344 μm (Fig. 2). Written informed consent was obtained from the patient for utilising his clinical details for this manuscript. Permission for using the patient data for this report was obtained from institutional review board and ethics committee (C/2019/06/03).

## Discussion and conclusion

Central serous chorioretinopathy is a hyperpermeable choroidal vascular disease characterised by serous detachment of the retina and/or retinal pigment epithelium (RPE) usually confined to the macula [11] In CSCR, the accumulation of SRF is mainly attributable to the imbalance between the increased choroidal vascular permeability and reduced SRF absorption by the RPE. The mechanisms for CSCR development following topical minoxidil therapy hypothesized are: 1) increased sympathetic activity leading to choroidal vasodilatation and choroidal vascular hyperpermeability 2) increased stimulation of VEGF causing increasing vascular permeability and 3) possible effects on the K-ATP channels on the RPE affecting its pumping mechanism and thereby leading to SRF accumulation [3, 5, 10] In our case, the choroidal vascular permeability changes were confirmed by ICGA and by increased choroidal thickness and pachy vessels seen on enhanced depth imaging OCT. Oral eplerenone in the treatment of CSCR has been suggested to work by decreasing the choroidal permeability and leakage through the blockage of the choroidal mineralocorticoid receptors [12, 13]. However, the drug’s efficacy in patients with CSCR was refuted by a recent study by Lotery et al. [14] A similar case of CSCR following topical 2% minoxidil application was reported by Scarinci et al. [9] In their case, the complete resolution of SRF was achieved after 1-month of stopping Minoxidil therapy. In our case, the SRF persisted despite the patient stopping Minoxidil therapy for 2 months. Also, a higher concentration 5% Minoxidil solution was used by the patient in our case. As the SRF was persistent despite stopping Minoxidil, we decided to start the patient on oral eplerenone therapy. In our case, the SRF resolved 1 month after starting therapy with oral eplerenone and remained stable even at 2-month follow-up visit. Given the latest publication which shows that eplerenone is not superior to placebo for CSCR, the improvement in our patient was either random or was related to a late contribution of stopping minoxidil. Despite the use of minoxidil by the patient and the other case report published in the literature, the relationship between minoxidil and CSCR still remains unproven. It is possible that this is only by chance.

To conclude, CSCR is a rare side-effect noted following prolonged topical minoxidil therapy for androgenic alopecia.

## Data Availability

The datasets used and/or analysed during the current study are available from the corresponding author on reasonable request.

## Abbreviations

VEGF: Vascular endothelial growth factor
CSCR: Central serous chorioretinopathy
OCT: Optical coherence tomography
ICGA: Indocyanine green angiography
SRF: Subretinal fluid
RPE: Retinal pigment epithelium
